# The EBI2 signalling pathway plays a role in cellular crosstalk between astrocytes and macrophages

**DOI:** 10.1038/srep25520

**Published:** 2016-05-11

**Authors:** Aleksandra Rutkowska, Sinead A. O’Sullivan, Isabelle Christen, Juan Zhang, Andreas W. Sailer, Kumlesh K. Dev

**Affiliations:** 1 Drug Development, School of Medicine, Trinity College, Dublin, Ireland; 2 Analytical Sciences and Imaging, Novartis Institutes for BioMedical Research, Novartis Pharma AG, Basel, Switzerland; 3 Developmental and Molecular Pathways, Novartis Institutes for BioMedical Research, Novartis Pharma AG, Basel, Switzerland

## Abstract

EBI2 is a G protein-coupled receptor activated by oxysterol 7α, 25-dihydroxycholesterol (7α25HC) and regulates T cell-dependant antibody response and B cell migration. We recently found EBI2 is expressed in human astrocytes, regulates intracellular signalling and modulates astrocyte migration. Here, we report that LPS treatment of mouse astrocytes alters mRNA levels of EBI2 and oxysterols suggesting that the EBI2 signalling pathway is sensitive to LPS-mediated immune challenge. We also find that conditioned media obtained from LPS-stimulated mouse astrocytes induces macrophage migration, which is inhibited by the EBI2 antagonist NIBR189. These results demonstrate a role for the EBI2 signalling pathway in astrocytes as a sensor for immune challenge and for communication with innate immune cells such as macrophages.

EBI2 (Epstein-Barr virus-induced gene 2, GPR183) is a G protein-coupled receptor that is expressed in peripheral blood mononuclear cells^1^,^2^,^3^ and activated by the oxysterol 7α25HC (7α, 25-dihydroxycholesterol)^4^. EBI2 regulates B cell positioning in lymphoid tissue and is crucial for launching T cell-dependant antibody response^5^,^6^. EBI2 is coupled exclusively to the G_i_ protein and when activated signals via two mitogen-activated protein (MAP) kinases, p38 and extracellular-signal-regulated kinase (ERK), as well as calcium and serum response element (SRE) transcription factor in a pertussis toxin (Ptx)-sensitive manner 1,3,7,8,9. Notably, it does not signal via nuclear factor of activated T cells (NFAT) or nuclear factor kappa B (NFκB)^7^,^8^. The most potent EBI2 agonist, 7α25HC, is synthesised from cholesterol by the enzymes cholesterol 25-hydroxylase (CH25H) and cytochrome P450 oxysterol 7-alpha-hydroxylase (CYP7B1) and is degraded by cholest-5-ene-3β,7α-diol 3β-dehydrogenase (HSD3B7)^4^,^10^. Upon challenge with lipopolysaccharide (LPS), B cells, macrophages, and other immune cells upregulate expression of EBI2 and the CYP7B1 and CH25H enzymes, while decreasing HSD3B7, thus apparently promoting EBI2 signalling^11^,^12^. It is noteworthy that such LPS immune challenge can also increase the levels of 25HC (the precursor to 7α25HC) in the humans and mice^11^,^13^ and moreover, that 25HC inhibits activity of human immunodeficiency virus, herpes simplex virus 1 and Ebola virus^14^. Thus, the induction of oxysterols in response to TLR4 stimulation or type I/II interferons might be a host strategy to fight such infections^11^,^15^, where EBI2 signalling may play a role.

The potential roles of the EBI2 signalling pathway molecules in disease and their potential uses as drug targets for Epstein-Barr virus (EBV) infection and EBV-mediated diseases as well as type-1-diabetes, multiple sclerosis, rheumatoid arthritis and systemic lupus erythematosus has been recently discussed^16^,^17^,^18^,^19^,^20^. For example, it has been shown that animals deficient in the 7α25HC synthesising enzyme, CH25H, exhibit worsened course of experimental allergic encephalomyelitis (EAE) and that CH25H knock-out (KO) macrophages have a proinflammatory phenotype compared to wild-type (WT)^21^. In agreement with the idea that EBI2 signalling may play a direct role in the central nervous system (CNS), we have reported that EBI2 is expressed in astrocytes, regulates astrocyte signalling, as well as astrocyte cell migration^22^. Another link between EBI2 signalling and brain disease comes from findings that CYP7B1/SPG5 mutations result in type 5 hereditary spastic paraplegia (hSPG5)^23^. Notably, in this illness, deficits in axonal trafficking, altered myelination state and neurodegeneration have been observed, suggesting a dysfunction in EBI2 signalling may induce brain pathophysiology as well as aberrant immune cell function. Whether mutations in CYP7B1 result in reduced levels of 7α25HC and subsequent reduced EBI2 activation in patients with hSPG5 and whether astrocytes play a specific role in this disease remains, however, to be clarified.

Here, to investigate further roles of the EBI2 signalling pathway in astrocytes, we examined its modulation by LPS in mouse astrocytes. We also examined if EBI2 signalling plays a role in the cellular communication of astrocytes with macrophages.

## Results

### LPS-stimulated mouse astrocyte conditioned media induces migration of macrophages

**Figure 1:**
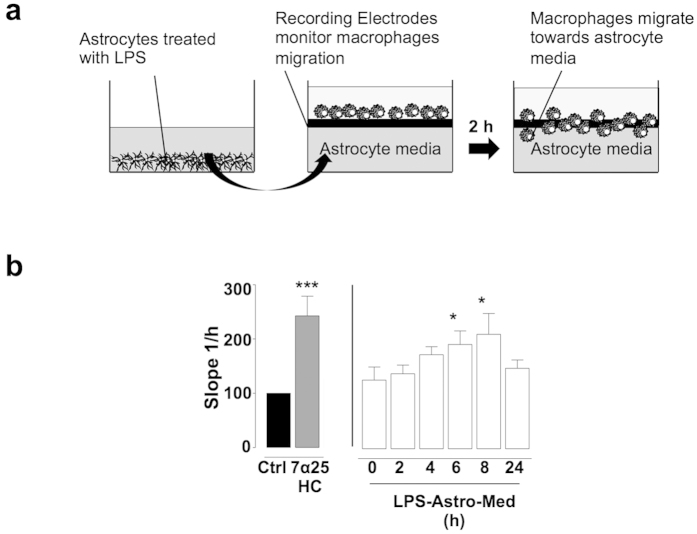
Conditioned media from LPS-treated mouse astrocytes induces migration of macrophage cells (RAW264.7). (**a**) Schematic of experimental setup. Transwell system where macrophages (RAW264.7) were plated on recording electrodes (upper chamber) and mouse astrocyte conditioned media with or without 7α25HC were added to the lower chamber. The migration of macrophages from the upper chamber to the lower chamber was measured by the recording electrodes two hours after addition of astrocyte media or drugs to the bottom chamber. (**b**) Treatment with 7α25HC (0.01 μM, 2 hours) induced a significant increase in macrophage migration. Astrocyte media collected after 6 and 8 hours of LPS (100 ng/ml) treatment induced macrophage (RAW264.7) migration while media from non-treated astrocytes or treated with LPS for 0, 2, 4, and 24 hours did not induce macrophage migration. Data presented as mean +/− SEM, n = 3 − 5, one-way ANOVAs with Dunnett’s post hoc tests, *p < 0.05, ***p < 0.001 vs. corresponding control.

There is a growing body of evidence demonstrating that astrocytes produce a number of signalling molecules (cytokines, chemokines, growth factors, nitric oxide and others) that can allow for communication with innate immune cells and perhaps promote their migration^24^. We first aimed to demonstrate that astrocytes stimulated with LPS could induce the migration of innate immune cells and, in this study, focused on their ability to regulate the migration of macrophages. Media supplemented with 7α25HC (0.01 μM) or taken from LPS (100 ng/ml, 0–24 h) treated mouse astrocytes (LPS-Astro-Med) was applied to the lower chamber of the xCELLigence^®^ migration transwell system containing macrophage RAW264.7 cells in the upper chamber (Fig. 1a). This LPS-Astro-Med induced significant migration of macrophage RAW264.7 cells in a time-dependent manner, compared to non-treated astrocytes (192.2% +/− 50.2% at 6 hours, 211.0% +/− 66.5% at 8 hours) (Fig. 1b). Notably, media from astrocytes treated with LPS for 2, 4 and 24 hours did not induce macrophage migration, indicating a temporal response and also indicating that the added LPS itself did not directly alter macrophage migration in these set of experiments (Fig. 1b).

### LPS regulates mRNA levels of the EBI2 signalling pathway in mouse astrocytes

**Figure 2:**
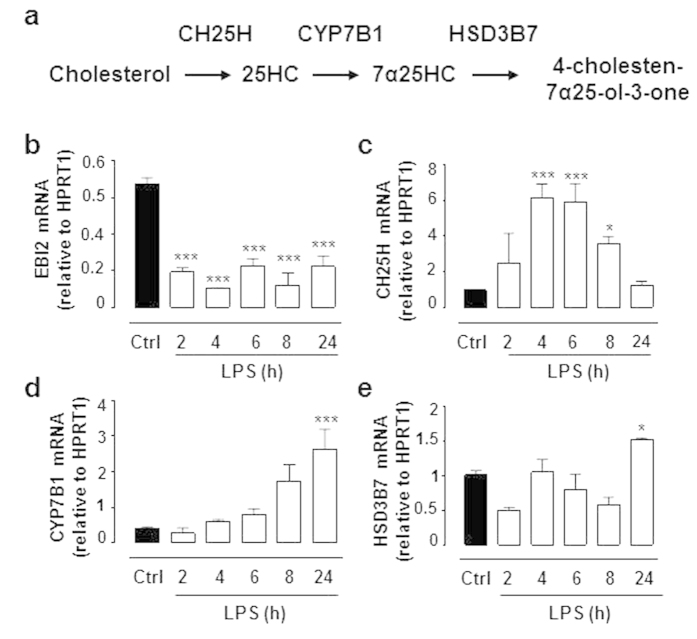
LPS modulates mRNA expression of molecules in the EBI2 signaling pathway in mouse astrocytes. (**a**) Synthetic and degrading pathway of 7α25HC. (**b**) Treatment of mouse astrocytes with LPS (100 ng/ml) induces a rapid and lasting suppression of EBI2 mRNA expression. (**c**) LPS induces CH25H mRNA expression in mouse astrocytes that peaks after four to six hours and declines thereafter. (**d**) LPS induces a slow and gradual increase in CYP7B1 mRNA expression in mouse astrocytes. (**e**) LPS induces increase in HSD3B7 mRNA expression after 24 hour treatment. Data presented as mean +/− SEM, n = 3, one-way ANOVA and Dunnett’s post-test, *p < 0.05, ***p < 0.001 vs. corresponding control.

Secondly, we aimed to determine whether mouse astrocytes respond to inflammatory insults (LPS) by regulating the EBI2 signalling pathway (receptor and signalling molecules). We and others have previously demonstrated that LPS alters mRNA levels of EBI2 and the 7α25HC synthesising and degrading enzymes (Fig. 2a)^11^,^12^,^25^. In accordance, here we found LPS suppressed EBI2 mRNA expression in mouse astrocytes (Fig. 2b). In addition, LPS induced expression of both 7α25HC synthesising enzymes namely CH25H and CYP7B1. The CH25H mRNA peaked after 4–6 hours (631.5% +/− 224.8% at 4 hours and 606.3% +/− 335.1% at 6 hours) (Fig. 2c). We also found LPS to increase CYP7B1 mRNA expression, with notable increases observed at 6 hours (187.7% +/− 120.9%) and 8 hours (421.5% +/− 312.9%), with significant increase at 24 hours (635.8% +/− 436.8%) (Fig. 2d). The mRNA levels of the 7α25HC degrading enzyme HSD3B7 remained constant for the first 8 hours of LPS treatment and increased after 24 hours (150.3% +/− 6.6% at 24 hours) (Fig. 2e). These results demonstrate that molecules of the EBI2 signalling pathway in astrocytes are sensitive to immune challenge such as LPS and support the idea that EBI2 may be involved in sensing infection in both immune and central nervous systems.

### LPS induces oxysterol release in mouse astrocytes

**Figure 3:**
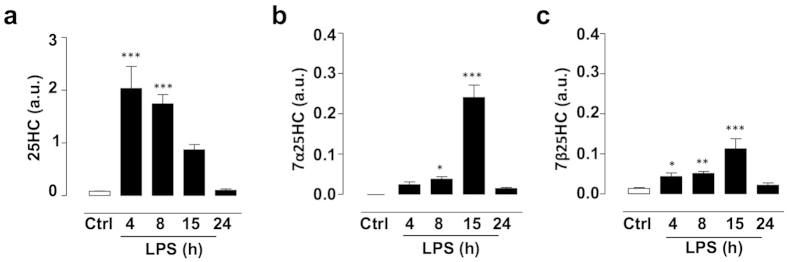
LPS induces oxysterol release in mouse astrocytes. Treatment of primary mouse astrocytes with LPS (100 ng/ml) induced levels of 25HC after four and eight hours (**a**) 7α25HC after eight and 15 hours (**b**) and 7β25HC after four, eight and 15 hours (**c**). Data presented as mean +/− SEM, n = 3 − 6. one-way ANOVAs with Dunnett’s post-tests, *p > 0.05, **p > 0.01, ***p > 0.001 vs corresponding control.

Thirdly, we examined if the LPS-induced changes in the 7α25HC synthesising and degrading enzymes were accompanied by altered levels of oxysterols in the astrocyte conditioned media. The levels of monohydroxylated oxysterols such as 25HC are induced in the CNS in human and animal subjects after treatment with LPS^11^,^13^. The release of these oxysterols has been attributed to immune cells, such as macrophages^11^,^12^,^13^. To demonstrate that astrocytes also produce oxysterols, mouse astrocytes were treated with LPS for 0–24 hours and levels of monohydroxylated (24SHC, 25HC and 27HC) and dihydroxylated (7α24SHC, 7α25HC, 7β25HC, 7α27HC and 7β27HC) oxysterols were measured with mass spectroscopy. The data showed no increase after LPS treatment in the levels of the following mono- and dihydroxylated oxysterols in the cell pellet: 24SHC, 7α24SHC, 27HC, 7α27HC, 7β27HC (data not shown). There was also no significant increase in the levels of mono- and dihydroxylated oxysterols (24SHC, 7α24SHC, 25HC, 7α25HC, 7β25HC, 27HC, 7α27HC and 7β27HC) in cell culture media after 0–24 h (data not shown). In contrast, LPS induced a significant increase in the levels of 25HC (the precursor to the EBI2 agonist 7α25HC) after four (2,435% +/− 1,224%) and eight (2,087% +/− 517%) hours of treatment in mouse astrocytes as detected in the cell pellets (Fig. 3a). The levels of the EBI2 agonist 7α25HC were also significantly induced in cell pellets after eight (4% +/− 1%) and 15 (24% +/− 5%) hours of LPS treatment (Fig. 3b). Likewise, levels of the closely related oxysterol 7β25HC were induced after LPS stimulation in the cell pellets after four (316% +/− 158%), eight (370% +/− 85%) and 15 (815% +/− 318%) hours (Fig. 3c). Together, this data, for the first time, demonstrates that astrocytes respond to the immune challenge by regulating EBI2 and oxysterol levels.

### EBI2 regulates macrophage migration induced by mouse astrocyte conditioned media

**Figure 4:**
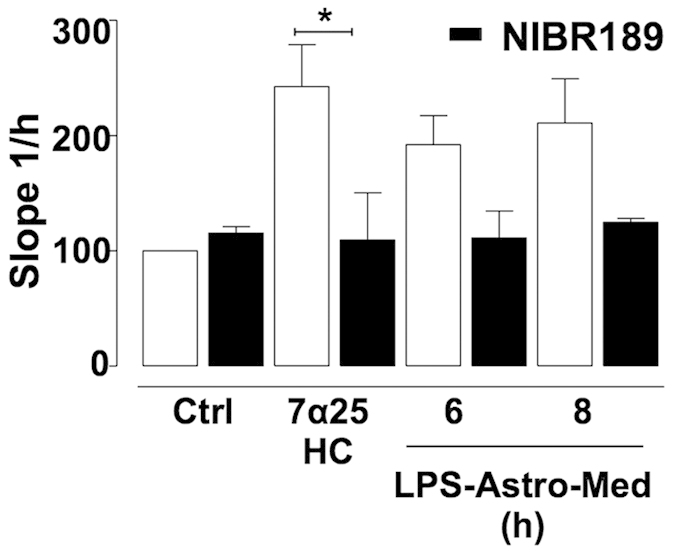
Macrophage (RAW264.7) migration induced by conditioned media from LPS-treated mouse astrocytes is reduced by EBI2 antagonism. Please see Fig. 1a for experimental setup. Both 7α25HC (0.01 μM) and astrocyte conditioned media obtained from 6 and 8 hour LPS-treated mouse astrocytes induced macrophage migration. Addition of the EBI2 antagonist (NIBR189, 10 μM) to 7α25HC or the LPS-treated mouse astrocyte media attenuated astrocyte conditioned media effects on macrophage migration. Data presented as mean +/− SEM n = 3, one-way ANOVAs with Bonferroni’s post-tests, *p < 0.05, vs. corresponding control.

Lastly, to investigate whether the observed migratory effects induced by LPS-Astro-Med were EBI2 dependant, the media was supplemented with the EBI2 antagonist (NIBR189 ref. [30], 10 μM). The effects of oxysterols on macrophage migration have been shown to be partially inhibited with EBI2 antagonist (NIBR189) indicating EBI2 mediated signalling^12^. In agreement with this previous study^12^, the EBI2 antagonist NIBR189 inhibited the effects of 7α25HC on macrophage migration (109.5% +/− 57.9%), that was exogenously applied to the lower chamber of the xCELLigence^®^ migration transwell system. Importantly, conditioned media taken from astrocytes stimulated with LPS for 6 and 8 hours (LPS-Astro-Med) and supplemented with EBI2 antagonist (NIBR189) attenuated the observed macrophage migration (Fig. 4). A trend decrease for this effect was observed suggesting partial EBI2 involvement in regulation of macrophage migration (Fig. 4). Overall, therefore, the data suggests that astrocytes stimulated with LPS can induce macrophage migration and, moreover, that EBI2 can regulate this cellular crosstalk.

## Discussion

Previous research has shown conflicting results on the role of CH25H in neuroinflammation. While Reboldi *et al*.^21^ showed that Ch25h deficient mouse macrophages adopt a pro-inflammatory phenotype and animals show a aggravated course of EAE, Chalmin and colleagues^26^ found that Ch25h deletion significantly attenuated EAE disease course by limiting trafficking of pathogenic CD4^+^ T lymphocytes to the central nervous system. The EBI2 signalling pathway (namely the receptor and 7α25HC and its metabolic enzymes) has also been shown to respond to immune challenge in primary human monocyte-derived macrophages^12^. In the current study, we investigated EBI2 signalling and function in mouse astrocytes and aimed to demonstrate the role of EBI2 in cellular communication between astrocytes and macrophages.

Here, we found that in response to LPS, primary mouse astrocytes significantly down-regulated mRNA expression of EBI2. Our data also indicated that mouse astrocytes regulated mRNA levels of the 7α25HC synthesising and degrading enzymes in response to a challenge with LPS. We can only speculate reasons for the relative slower temporal activations of CYP7B1 and HSD3B7 compared to CH25H activation, which at present remains unclear. Importantly, this data is consistent with the mRNA expression of these enzymes in macrophages treated with LPS showing that CH25H expression peaks after two hours, CYP7B1 mRNA can be observed over time of LPS treatment and CYP27A1 and HSD3B7 transcripts are down regulated within two or six hours^12^.

We found that upregulation of the synthesising enzymes was accompanied by an upregulation of 7α25HC levels in mouse astrocyte conditioned media following LPS treatment. The findings that LPS increased the levels of 7α25HC in mouse astrocyte conditioned media was associated with the ability of this media to promote migration of macrophages. We also found that conditioned media obtained from LPS-stimulated astrocytes induced macrophage migration that was partially attenuated with the EBI2 antagonist NIBR189. Thus, regulation of the EBI2 signalling pathway (both the receptor and enzymes that control levels of the ligand) in astrocytes appears to promote the migration of macrophage cells.

Taken together, the data indicates that molecules in the EBI2 signalling pathway are sensitive to pro-inflammatory signals, and that EBI2 plays a role in communication between astrocytes and macrophages.

## Methods

### Cell Culture

All animal procedures were approved by the institutional ethics committee (Novartis Pharma, Basel Switzerland and Trinity College Dublin, Ireland). Methods were carried out in accordance with the approved guidelines. Mouse astrocytes^27^,^28^,^29^,^30^ and mouse macrophage cells (RAW264.7)^12^ were cultured as we have described before. Prior to stimulation, astrocytes were starved in serum-free media for 4 hours. Astrocytes were then treated with LPS (100 ng/ml, Sigma) with or without the EBI2 antagonist (NIBR189, Novartis)^31^ and/or 7α25HC (prepared as 10 mM stock in 90% DMSO).

### Real Time Quantitative Polymerase Chain Reaction (RT-qPCR)

For mRNA expression analysis, after treatment, astrocytes were washed twice with PBS, and either directly scraped or the cell pellet was resuspended in RA1 lysis buffer (Machery-Nagel, 740961) supplemented with 10% β-mercapthoethanol and frozen at −80 °C. RNA was isolated using the RNeasy Mini Kit (Qiagen Hilden, Germany). After reverse transcription of mRNA (10 min, 25 °C; 120 min, 37 °C; 5 sec, 85 °C) using the High Capacity cDNA Reverse Transcription Kit (Applied Biosystems, Darmstadt, Germany), qRT-PCR was performed with the 7900HT Fast Real-Time PCR System (Applied Biosystems) according to the Standard Thermal Cycler Protocol (2 min, 50 °C; 10 min, 95 °C; 40 cycles 15 sec, 95 °C and 1 min, 60 °C). TaqMan Gene Expression Assays used the following FAM dye-labeled TaqMan mouse probes: EBI2 (Mm02620906-s1), CH25H (Mm00515486-s1), CYP7B1 (Mm00505894-g1), HSD3B7 (Mm01545399), HPRT1 (Mm01545399-m1) (Applied Biosystems). Applied Biosystems provide the context sequence (a 25 nucleotide sequence within which the probe sequence will lie) and within the details section of each of the assays provide a RefSeq, assay location nucleotide and amplicon length. For each of these assays the above mentioned context sequence can be found by counting 12 nucleotides on either side of the assay location nucleotide on the RefSeq (within this stretch of 25 nucleotides). The amplicon context sequence can similarly be found by counting the number of nucleotides corresponding to the amplicon length on either side of the assay location nucleotide on the RefSeq. Within this stretch of nucleotides on the Refseq the amplicon is located. Each condition was run in quadruplicates, with each quadruplicate RNA sample run in duplicates and the experiments were repeated 3 times. The threshold was set manually for all samples. The analysis was performed with the SDS 2.3 software. The relative expression of EBI2, CH25H, CYP7B1 and HSD3B7 to the reference gene (HPRT1) was determined.

### Ultra-high performance liquid chromatography and tandem mass spectrometry (UHPLC-MS/MS)

For mass spectroscopy the oxysterol standards and internal standards were purchased from Avanti Polar Lipis: 25HC, 27HC, 24SHC, 25HC-d6, 27HC-d6, 24(R/S)HC-d7, 7α25HC, 7α27HC, 7α24SHC, 7β25HC, 7β27HC, 7α25HC-d6, 7α27HC-d6, 7α24(R/S)HC-d7. Butylated hydroxytoluene (BHT) was purchased from SAFC (Sigma). Thirty million cells in 1 ml of H_2_O containing a mixture of deuterated internal standard compounds and 200 μM BHT were lysed using a Precellys^®^24 (Bertin Technologies, France) before extraction. To extract the oxysterols the lysed cells or 1 ml culture media were gradually mixed with 9 ml of EtOH by shaking and 4 °C cooling to allow slow precipitation. The extracts were then dried under N_2_ steam at 40 °C. The residue was resuspended in 100μl EtOH/H_2_O and 10 μl were injected onto a reverse-phase column for the ultra-high performance liquid chromatography and tandem mass spectrometry (UHPLC-MS/MS) analyses of oxysterols. The analyses were carried out on a Nexera UHPLC system (Shimadzu, Japan) coupled to a QTrap^®^6500 (ABSciex, Framingham, USA) mass spectrometer. Chromatographic separation was achieved using an Acquity UPLC^®^ BEH C18 column (100 × 2.1 mm, particle size 1.7 μm) with a VanGuard™ pre-colum Acquity UPLC^®^ BEH C18 (5 × 2.1 mm, particle size 1.7 μm) (Waters Assoc., USA). For the separation of monohydroxylated cholesterols, the mobile phases were delivered at a flow rate of 600 μl and a column temperature of 55 °C. Mobile phase A consisted of 5% MeOH in H_2_O and 0.1% formic acid (FA), whereas phase B of MeOH/acetonitrile (ACN) and 0.1% FA. The gradient program was composed of three isocratic steps: 6 min, 65% in phase B followed by 7 min, 70% in phase B and 2 min, 97% in phase B. For the separation of the dihydroxylated cholesterols, a 10 min linear gradient program from 73–97% in phase B was applied with a flow rate of 400 μl/min and a column temperature of 55 °C. Mobile phase A consisted of 5% MeOH in H_2_O and 0.1% FA whereas phase B of MeOH and 0.1% FA. The mass spectrometer was equipped with an electrospray ionization source. The instrument was operated in a positive ion mode. Following MS/MS transitions were applied for the detection of oxysterols: m/z 385 → m/z 157 and 159 for the mono-hydroxylated cholesterols and m/z 383 → m/z 157 and 159 for dihydroxylated cholesterols.

### Migration Assays

Migration of mouse macrophages (RAW264.7) was conducted at 37 °C and 5% CO_2_ in a humidified incubator using the xCELLigence® directed migration platform (Roche Applied Biosciences) with 16-well CIM-plates as we have described previously^22^. In brief, the migration of cells through the electrode array from the upper to lower chambers is measured by an increase in impedance. The bottom chamber of the plate was filled with either astrocyte conditioned media or serum free media supplemented with 7α25HC (0.01 μM) with or without EBI2 antagonist NIBR189 (10 μM). Cell migration was recorded immediately after the plate was re-inserted and recorded for 2 hours.

## Additional Information

**How to cite this article**: Rutkowska, A. *et al.* The EBI2 signalling pathway plays a role in cellular crosstalk between astrocytes and macrophages. *Sci. Rep.* **6**, 25520; doi: 10.1038/srep25520 (2016).
